# Early Postnatal EEG Features of Perinatal Arterial Ischaemic Stroke with Seizures

**DOI:** 10.1371/journal.pone.0100973

**Published:** 2014-07-22

**Authors:** Evonne Low, Sean R. Mathieson, Nathan J. Stevenson, Vicki Livingstone, C. Anthony Ryan, Conor O. Bogue, Janet M. Rennie, Geraldine B. Boylan

**Affiliations:** 1 Neonatal Brain Research Group, Irish Centre for Fetal and Neonatal Translational Research, Department of Paediatrics and Child Health, University College Cork, Cork, Ireland; 2 Elizabeth Garrett Anderson Institute for Women's Health, University College London Hospital, London, United Kingdom; University Medical Center Groningen UMCG, Netherlands

## Abstract

**Background:**

Stroke is the second most common cause of seizures in term neonates and is associated with abnormal long-term neurodevelopmental outcome in some cases.

**Objective:**

To aid diagnosis earlier in the postnatal period, our aim was to describe the characteristic EEG patterns in term neonates with perinatal arterial ischaemic stroke (PAIS) seizures.

**Design:**

Retrospective observational study.

**Patients:**

Neonates >37 weeks born between 2003 and 2011 in two hospitals.

**Method:**

Continuous multichannel video-EEG was used to analyze the background patterns and characteristics of seizures. Each EEG was assessed for continuity, symmetry, characteristic features and sleep cycling; morphology of electrographic seizures was also examined. Each seizure was categorized as electrographic-only or electroclinical; the percentage of seizure events for each seizure type was also summarized.

**Results:**

Nine neonates with PAIS seizures and EEG monitoring were identified. While EEG continuity was present in all cases, the background pattern showed suppression over the infarcted side; this was quite marked (>50% amplitude reduction) when the lesion was large. Characteristic unilateral bursts of theta activity with sharp or spike waves intermixed were seen in all cases. Sleep cycling was generally present but was more disturbed over the infarcted side. Seizures demonstrated a characteristic pattern; focal sharp waves/spike-polyspikes were seen at frequency of 1–2 Hz and phase reversal over the central region was common. Electrographic-only seizure events were more frequent compared to electroclinical seizure events (78 *vs* 22%).

**Conclusions:**

Focal electrographic and electroclinical seizures with ipsilateral suppression of the background activity and focal sharp waves are strong indicators of PAIS. Approximately 80% of seizure events were the result of clinically unsuspected seizures in neonates with PAIS. Prolonged and continuous multichannel video-EEG monitoring is advocated for adequate seizure surveillance.

## Introduction

Perinatal arterial ischaemic stroke (PAIS) occurs approximately 1 in 2500 livebirths and is recognized as a common cause of early onset neonatal seizures. [1] Approximately 20% of neonatal seizures are due to PAIS, [2] and neonatal seizures have been noted in up to 26% of neonates with PAIS. [3] Generally, neonates with PAIS are non-encephalopathic but those with significant seizure burden can be neurologically abnormal, making the distinction from seizures due to other causes such as hypoxia-ischaemia difficult in the acute neonatal period. [4] The diagnosis of PAIS should be suspected when seizures are observed in non-encephalopathic neonates within the first 48 hours of birth. [5] While cranial ultrasound scans have been shown to have good diagnostic capabilities when performed after day 4, [6] confirmation of diagnosis is only reliably achieved with magnetic resonance imaging (MRI); however this facility is not readily available in many institutions.

Electroencephalogram (EEG) or amplitude integrated-EEG (aEEG) is now one of the first diagnostic tools available at the cotside in the neonatal intensive care unit for the assessment of cerebral function. Most studies in PAIS have described EEG changes in the first week after birth, but typical changes observed in the first 48 hours after birth have not been described. Early EEG may distinguish neonates with PAIS from those with hypoxic-ischaemic encephalopathy (HIE) [3] and other aetiologies, providing invaluable support for clinical decision-making and counselling. The aEEG has been used to obtain additional information in neonates with PAIS by van Rooij *et al.* [7] and Mercuri *et al.* [8]; however these studies have not given details on the characteristics of electrographic seizures. Early accurate recognition of PAIS would be helpful in distinguishing neonates with seizures who do not fulfil the current criteria for therapeutic hypothermia, but who require thrombophilic screening and high quality MRI for diagnosis and prognosis. The aim of our study was to characterize the early postnatal EEG findings in term neonates with PAIS who had seizures.

## Methods

### Ethics statement

This study was approved by the Clinical Research Ethics Committees of the Cork Teaching hospitals, Ireland and the National Health Service in the United Kingdom (UK), via the Integrated Research Application Service. Written, informed consent was obtained from at least one parent of each neonate who participated in this study.

### Patients

Neonates were enrolled from Cork University Maternity Hospital (CUMH), Ireland between June 2003 and October 2011 and University College London Hospital (UCLH), UK from January 2009 to October 2011 as part of an ongoing study of neonatal seizures. Neonates >37 weeks gestation were enrolled for EEG monitoring if they fulfilled at least one of the following criteria: Apgar score <6 at five minutes; a continued need for resuscitation after birth; any clinical evidence of encephalopathy or seizures within 72 hours of age. The diagnosis of PAIS was based on neuroimaging evidence of focal infarction affecting at most two arterial territories. Study analysis included only neonates with PAIS who had electrographic seizures. Neonates with HIE, infections, inborn errors of metabolism, blood disorders, venous or multiple infarctions were excluded due to differing pathogeneses and clinical manifestations when compared to those with focal arterial infarction.

### Clinical features

All clinical seizures were treated as well as seizures recognized by the clinical team interpreting the aEEG. The aEEG used to confirm suspected seizures was also used as an aid in clinical decision-making at the cotside. Concern regarding any abnormal behaviour or aEEG pattern prompted a review of the multichannel EEG from the neurophysiologist in each hospital. Immediate reporting of the multichannel EEG was not always available; the aEEG and clinical suspicion were the mainstays of seizure diagnosis. Phenobarbitone was the first-line anticonvulsant administered to a maximum dose of 40 mg/kg intravenously. Second-line anticonvulsants were administered if clinical and/or electrographic seizures recurred following phenobarbitone administration. In both hospitals, second-line anticonvulsant was either intravenous phenytoin or midazolam. Although standardized protocols for the use of anticonvulsants were similar in both hospitals, the choice of second-line anticonvulsant administration was at the discretion of the attending neonatologist. The timing and dose of each anticonvulsant as well as morphine administered were recorded in all neonates.

### EEG features

Clinical details of all neonates were obtained at the time of monitoring. Throughout the study, EEG recording methods were identical at both hospitals. A Nicolet monitor (Carefusion NeuroCare, Wisconsin, USA) was used to record multichannel video-EEG, using the 10–20 system of electrode placement modified for neonates. [9] EEG monitoring was commenced when recruitment criteria were met and continued for at least 20 hours. Scalp electrodes were placed at F3, F4, C3, C4, T3, T4, O1, O2 and Cz locations to record EEG activity from the frontal, central, temporal and occipital areas. Parietal electrodes (P3–P4) were also used where possible. Impedances below five kΩ were maintained. Simultaneous bilateral aEEG trends, electrocardiogram and respiration traces were also displayed on the monitor.

All EEG recordings from each neonate were independently reviewed by an experienced neonatal electroencephalographer (GBB). The entire background EEG pattern was graded and assessed for continuity, symmetry, synchrony and other specific features. Sleep cycling was assessed as being present, absent or disturbed in each neonate; a disturbed sleep cycling signified an interruption to the expected sleep cycle architecture of healthy term neonates. [10] Significant EEG suppression was defined as EEG activity below five µV in all EEG channels for at least 10 seconds respectively. The morphology of seizures was also assessed. An electrographic seizure was defined as a sudden and evolving repetitive stereotyped waveform with a definite start, middle and end, lasting for at least 10 seconds [11] on at least one EEG channel. Status epilepticus was defined as continuous or accumulative electrographic seizure activity lasting ≥50% of a one-hour period. [12]

Any associated clinical correlates with all electrographic seizures annotated were analyzed using the simultaneous video recording. Electrographic-only seizures were defined as clear electrographic seizures without any clinical correlates. [13] Electroclinical seizures were defined as electrographic seizures accompanied with behavioural correlates. Clinical seizures were defined as paroxysmal alterations in neurological function (behaviour, motor or autonomic); the description was based on those categorized by Volpe. [2] Subtle seizures were defined as paroxysmal behaviours (including changes in autonomic parameters) which were not clearly clonic, tonic or myoclonic seizures [2] and included behaviours such as eye blinking, pedalling or cycling movements of the limbs, hiccups, sucking or chewing movements and apnoeic spells.

### Radiographic features

MRI studies were performed in a Siemens Avanto 1.5 Tesla unit (Siemens Ag, Erlangen, Germany) and CT scanning was performed using a Toshiba Aquilion 4-detector row CT (Toshiba, Tochigi-ken, Japan). All imaging studies were performed without sedation. Neonates were transferred to the MRI scanner in an MRI-compatible incubator with integrated neonatal array coils (MR Diagnostics Incubator, Lammers Medical Technology GmbH, Luebeck, Germany). The arterial territory and estimated size of cerebral infarction based on methods described by Marks *et al*., [14] were reported by an experienced paediatric radiologist (COB).

### Statistical analysis

The total seizure burden was defined as the total duration of recorded electrographic seizures in minutes. Electrographic seizure window was defined as the timepoint between the first and last recorded electrographic seizure in hours. Seizure burden was also expressed in terms of seizure per hour and was calculated using a formula:

Seizure burden =  total seizure burden (minutes)/ electrographic seizure window (hours).

In each neonate, the mean seizure duration is calculated as the proportion of the total seizure burden in seconds relative to the number of seizures.

Mean seizure duration  =  total seizure burden (in seconds) / total number of seizures.

To avoid neonates with many seizures having much influence on the results, summary measures were calculated for each neonate. These summary measures were percentages of the number of seizure events and the seizure burden (seizure duration in minutes) associated with electrographic-only, electroclinical seizures and the duration when viewing of the video was obscured (for example during a medical procedure); they were calculated relative to the total number of electrographic seizures and the total seizure burden (seizure duration in minutes). For example:

% number of electrographic-only seizures =  (the number of electrographic-only seizures/ the total number of seizures) * 100

% seizure burden of electrographic-only seizures =  (the seizure burden of electrographic-only seizures/ the total seizure burden) * 100

% number of electroclinical seizures =  (the number of electroclinical seizures/ the total number of seizures) * 100

% seizure burden of electroclinical seizures =  (the seizure burden of electroclinical seizures/ the total seizure burden) * 100.

These summary measures were then described across all neonates using medians and interquartile ranges (IQR). For paired comparisons, the Wilcoxon signed-rank test was used. All statistical analyses were performed using SPSS Statistics 20.0 (IBM SPSS Statistics, Illinois, USA). All tests were two-sided; p-value <0.05 was considered to be statistically significant.

## Results

During the study, nine neonates with PAIS who had continuous early EEG monitoring had electrographic seizures. Five neonates had coagulation testing and none had thrombophilic disorders. Table 1 lists the clinical demographics and outlines the MRI findings in eight of the nine neonates with various degrees of middle cerebral artery (MCA) infarction; one neonate had CT imaging. Cranial imaging was undertaken at a median (IQR) of 5 (3–12) days after birth.

**Table 1:** Demographics and neuroimaging features of neonates in the order of increasing seizure burden.

Neonate	1	2	3	4	5	6	7	8	9
Birthweight (grams)	3700	3740	3750	3410	2830	3420	3160	3670	3480
Gestation (weeks)	40	39	41	41	39	41	39	41	41
Gender	Male	Male	Male	Female	Female	Female	Male	Male	Male
Perinatal events	None	Polyhydramnios, PROM (36 h)	NRCTG	None	NRCTG, PROM (>18 h)	None	NRTCG	FTP	FTP
Mode of delivery	VV	VV	EMCS	Forceps	EMCS	Ventouse	EMCS	EMCS	EMCS
First pH	7.42	7.04	7.13	7.29	7.00	7.34	7.30	7.41	7.27
5 min Apgar	10	9	9	10	6	7	10	10	10
Age at first clinical seizure (hours)	36	54	20	6	47	33	15	18	33
First clinical seizure	RUL	Dusky episodes	LS	RS	LLL	RS	RUL	RUL	LUL
Age at EEG (hours)	54	59	26	9	53	3	18	19	36
Age at first recorded EEG seizure (hours)	54	60	26	9	53	39	19	19	36
EEG duration (hours)	25	70	49	39	44	46	49	63	229
Cerebral infarction	LMCA	LMCA	RMCA	LMCA, RMCA	RMCA	LMCA	LMCA	LMCA, LPCA	RMCA
Age at cranial imaging (days)	5	8	29	3	10	3	2	3	14
Estimated size of infarction (%)	<33	>66	<33	<33	33–66	33–66	<33	>66	33–66

EMCS, emergency Caesarean section; FTP, failure to progress; LLL, left lower limb clonic; LMCA, left middle cerebral artery; LPCA, left posterior cerebral artery; LS, left-sided clonic; LUL, left upper limb clonic movements; NRCTG, non-reassuring cardiotocogram; PROM, premature rupture of membranes; RMCA, right middle cerebral artery; RS, right-sided clonic; RUL, right upper limb clonic; VV, vertex vaginal.

**Figure 1:**
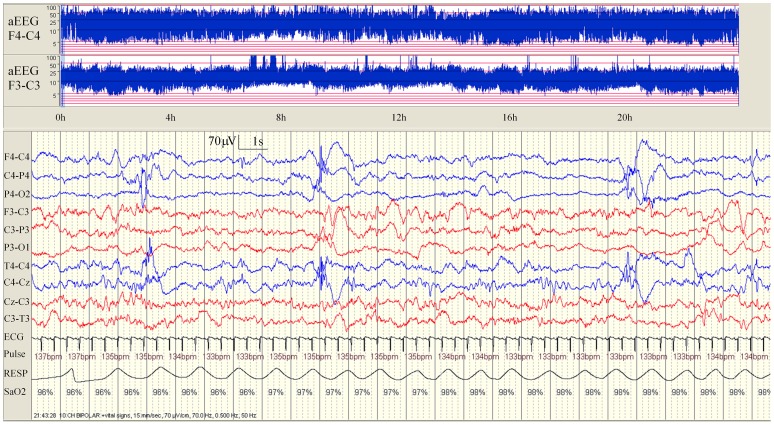
Background EEG pattern in a neonate (case 9) with a right middle cerebral artery infarction. Note the mild voltage reduction over the right hemisphere on EEG (blue channels) which is also evident on the aEEG with a wider band on the right in comparison to the left side. In addition, intermittent right-sided bursts of higher voltage sharpened theta activity are also evident. Some sleep cycling is also present over the left albeit disturbed but this is absent over the right side.

**Figure 2:**
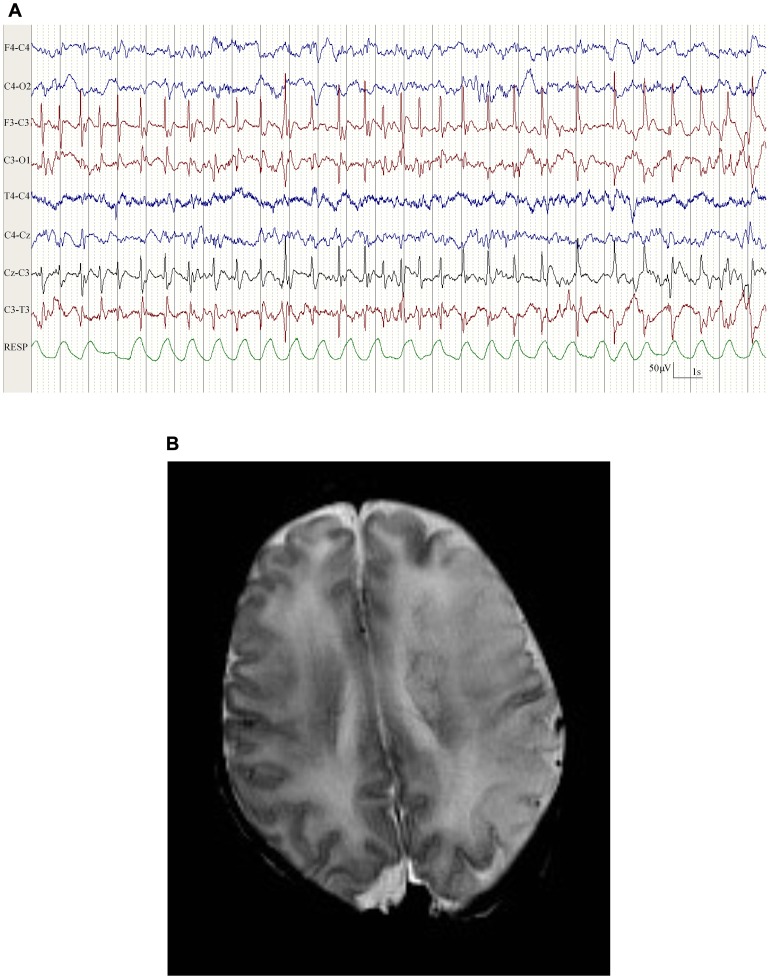
A. EEG in a neonate (case 6). Seizures arising from the left hemisphere corresponding with a left middle cerebral artery infarction on cranial MRI. **B. Cranial MRI in a neonate (case 6).** The sequence is an axial T2 turbo spin echo performed on day 7 of life. Note the characteristic focal spike and wave discharges over the left hemisphere with phase reversal over the left central region.

Table 2 summarizes the background EEG and seizure characteristics for each neonate. In all neonates, a continuous background pattern was present but voltage suppression and intermittent sharp theta discharges were seen over the infarcted side (figure 1). Background EEG suppression was greatest in cases where the estimated size of infarction was larger than 66% of one hemisphere. Sleep cycling was present in all cases but disturbed in 5 of the 9 neonates. The morphology of seizures in neonates with PAIS showed a characteristic pattern in all cases (figure 2). Spike and polyspike waves at a frequency of 1–2 Hz were seen over the infarcted side and phase reversal of these spikes over the central region was evident as the seizure evolved. Higher frequency temporal discharges were seen during apnoea in a neonate who presented with dusky episodes.

**Table 2:** Characteristics of EEG and seizures.

Neonate	1	2	3	4	5	6	7	8	9
Summary of background EEG feature									
Continuous activity	Yes	Yes	Yes	Yes	Yes	Yes	Yes	Yes	Yes
Symmetry	Left mild suppression	Left significant suppression	Right mild suppression	Good	Right mild suppression	Good	Good	Left significant suppression	Right mild suppression
Intermittent features	Left-sided sharp theta bursts	Left-sided theta sharp waves	Right focal sharp waves	Left-sided sharp waves in quiet sleep	Right-sided theta sharp waves	Left-sided theta sharp waves	Left-sided focal sharp theta waves	Left-sided theta sharp waves	Right-sided sharp waves
Sleep cycling	Normal bilaterally	Disturbed unilaterally	Disturbedbilaterally	Disturbed bilaterally	Normal bilaterally	Normal bilaterally	Disturbed unilaterally	Disturbed unilaterally	Normal bilaterally
Seizure morphology	Focal spikes over left central with phase reversal	Focal spikes over left central with phase reversal	Focal spikes over right central with phase reversal	Focal spikes & polyspikes over left central with phase reversal	Focal spikes & polyspikes over right central with phase reversal	Focal spikes over left central with phase reversal	Focal spikes over left central with phase reversal	Focal spikes & polyspikes over left central with phase reversal	Focal spikes & polyspikes over right central with phase reversal
Summary of seizure burden									
Total seizure burden (minutes)	19	67	101	133	162	201	266	327	332
Seizure burden (minutes/hour)	2.70	7.28	27.60	5.53	10.27	18.15	12.77	9.25	6.18
Mean seizure duration (seconds)	370	98	356	362	120	523	143	195	146
Seizure window (hours)	7	9	4	24	16	11	21	35	54
Status epilepticus	None	None	Yes	None	None	Yes	Yes	Yes	Yes
Number of seizures (n)	3	41	17	22	81	23	112	101	136
Seizure classification									
Electrographic-only seizures: n (%)	0 (0)	27 (66)	8 (47)	20 (91)	77 (95)	13 (57)	77 (69)	62 (61)	121 (89)
Electrographic-only seizure burden: minutes (%)	0 (0)	28 (42)	26 (25)	129 (97)	146 (90)	74 (37)	129 (49)	244 (74)	282 (85)
Electroclinical seizures: n (%)	2 (66)	10 (24)	7 (41)	1 (4.5)	3 (3.7)	9 (39)	32 (29)	35 (35)	15 (11)
Electroclinical seizure burden: minutes (%)	18 (95)	30 (44)	48 (48)	3 (2)	15 (9)	108 (54)	126 (47)	80 (24)	50 (15)
Clonic/subtle seizures: n	2/0	0/10D	5/2C	1/0	3/0	0/9S	17/15S	16/19Y	9/6M
Video obscured: n (%)	1 (33)	4 (10)	2 (12)	1 (4.5)	1 (1.3)	1 (4)	3 (2)	4 (4)	0 (0)

Subtle seizures: C, cycling movements of the limbs; D, desaturations; M, mouthing and smacking; S, sucking; Y, yawning.

Of 536 electrographic seizures identified from multichannel EEG in this cohort of neonates with PAIS; 519 were classified (table 2). Accumulatively, there were more electrographic-only seizure events (n = 405; 78%) than electroclinical seizure events (n = 114; 22%). Summary measures of each neonate showed that the median (IQR) electrographic-only seizure events was higher than electroclinical seizure events [66 (52–90) *vs* 29 (8–40)%; p = 0.051]. Subtle seizures were noted in six of nine neonates and manifested activities such as pedalling or cycling movements of the limbs, sucking or chewing movements. Other occasional subtle seizures noted were hiccups and eye blinking episodes. When electroclinical seizures were subdivided, there were more subtle (n = 61; 12%) than clonic seizures (n = 53; 10%) [median (IQR) of subtle *vs* clonic seizures = 12 (0–22) *vs* 7 (2–24)%; p = 0.553]. The median percentage of seizure burden of electrographic-only was higher than electroclinical seizures [49 (31–88) *vs* 44 (12–51)%; p = 0.515]. This is despite the significantly shorter median duration of electrographic-only when compared to electroclinical seizures [100 (55–173) *vs* 181 (95–359) seconds; p<0.001].

**Figure 3:**
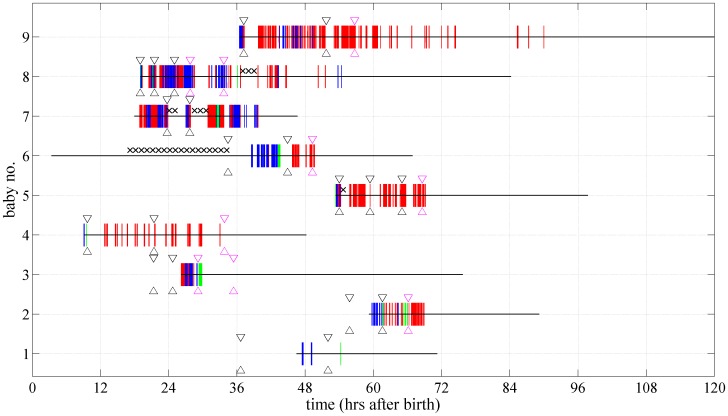
Characteristics of seizures and anticonvulsant administration in each neonate. Vertical red lines denote the presence of electrographic-only seizures, vertical blue lines denote electroclinical seizures and vertical green lines denote obscured seizures. Horizontal black line denotes the period of EEG monitoring. Black crosses denote missing data. Timepoints bounded by black arrows denote the first-line anticonvulsant administration while the magenta arrows denote the second-line anticonvulsant administration.

The temporal distribution of electrographic-only and electroclinical seizures with anticonvulsant administration superimposed for each neonate are shown in figure 3. In four of nine neonates (cases 1, 2, 3 and 6), anticonvulsants were administered prior to prolonged multichannel EEG monitoring, hence before the first electrographic seizure. All nine neonates with PAIS received first-line anticonvulsants at 34 (20–46) hours while seven neonates received second-line anticonvulsants at 48 (29–66) hours.

## Discussion

The background EEG generally showed suppression over the affected side; this was quite marked (>50% amplitude reduction) if the infarction was large. Characteristic unilateral theta bursts with intermixed sharp or spike waves were seen in all cases over the infarcted side. Sleep cycling was generally present but was more disturbed over the infarcted side. Seizures in neonates with PAIS appear to have a characteristic pattern and in all cases, focal sharp waves/spike-polyspike seizure discharges were seen at a frequency of 1–2 Hz over the area of infarction. In our experience, the morphology of these seizures is quite characteristic and markedly different from seizures due to HIE. [15] All neonates in our series had MCA involvement; seizures were generally seen over the central region and phase reversal of spike and polyspike discharges were a common finding. This is the first study to describe these characteristic EEG findings in a series of neonates with PAIS in the early postnatal period; these findings may prove very useful for early diagnosis of neonates with seizures.

Indeed PAIS tends to be a clinical diagnosis when three important findings are present: no clear history of HIE, seizure onset beyond 12 hours after birth and focal seizures. In many instances when the affected cases are discussed retrospectively, subtle details are often missed; they usually revealed a slightly complicated antenatal history such as mild changes on the cardiotocogram or meconium stained delivery. [16] Apgar scores and clinical history may be subjective. We advocate the use of the EEG as an adjunct to suggest the early diagnosis of PAIS during the neonatal period when clinical suspicions are aroused.

Comparing one-channel with the two-channel aEEG recordings in 34 neonates who had seizures due to unilateral brain injury, van Rooij *et al.* showed more varied seizures patterns, asymmetry in the background activity and a difference in sleep cycling on the ipsilateral side, [7] however this study gave no specific analysis on a subgroup of neonates who had PAIS (n = 5) or specifically those who had MCA involvement (n = 3). Using a four-channel aEEG in 19 neonates with PAIS (6 neonates with asymmetrical and 2 with bilateral sharp waves/ spikes, 8 no seizures, 3 not recorded), Mercuri *et al.* showed that the presence of seizures accompanied by a normal background EEG was not related to abnormal outcome; [8] this indicates that both factors are poor predictors of outcome. Although our study was not aimed to assess outcome, we believe that an abnormal background and the presences of seizures have a much higher prognostic value. Also, the study by Mercuri *et al.* had not assessed seizures as an independent factor in determining outcome. [8] Multichannel EEG has been shown to be more accurate than the aEEG in detecting seizures. Our EEG findings based on multichannel EEG recordings are similar to studies by van Rooij *et al.* [7] and Mercuri *et al.* [8] which used the aEEG, however we have provided more details on the characteristics of seizures early in the neonatal period in terms of seizure morphology and more detailed seizure characteristics in a cohort of neonates with PAIS.

Several studies have reported the electrographic seizure burden in neonates with HIE, [17], [18] but none has quantified seizure burden in neonates with PAIS using continuous multichannel EEG. A study by Rafay *et al.* compared the EEG characteristics between neonates with PAIS and HIE; [3] they showed that there was no significant difference in the number of neonates who had electrographic seizures (PAIS *vs* HIE: 7/27 *vs* 13/35; p = 0.350]. Although their study contributed further to our understanding of neonatal seizures, the results were limited because EEG findings were described exclusively from EEG reports generated by a neurophysiology service. In our study, we have explored further on the multichannel EEG recordings. The overall seizure burden was high in our study; prolonged multichannel video-EEG monitoring showed that the number of seizures is higher than clinically apparent. In our study, anticonvulsants were administered when there was a clinical concern of seizures. The use of anticonvulsants may have resulted in more electrographic-only seizures [19]; and in our study we have shown that 80% of seizure events were electrographic-only seizures. The high number of seizures which we uncovered in this group of neonates was surprising but reinforces the need for early and continuous EEG monitoring in this group of neonates. In comparison, electroclinical dissociation has been reported to occur up to 28% of neonates with HIE; however this figure was based on aEEG findings in neonates above 32 weeks gestation and its association with anticonvulsant administration was not described. [20] The studies by van Rooij *et al.* [7] and Mercuri *et al.* [8] did not provide information on the dissociation of seizures. Many of the previous studies reported the clinical response to anticonvulsants without any EEG monitoring. [21]–[23] It is known that anticonvulsants can be a sedative agent and lead to electroclinical uncoupling or dissociation. [24] Clinical seizures are therefore a poor indicator when it comes to assessing the response to anticonvulsants; hence the true response of anticonvulsants in seizure control in neonates with PAIS remains unknown. Our study highlights that despite the use of anticonvulsants, under tight EEG monitoring, there are still ongoing electrographic seizures in neonates with PAIS. Neonatologists should be aware of this when treating neonates with PAIS who are already treated with initial anticonvulsants, particularly in the absence of EEG monitoring. This also explains why several neonates in our study had many hours of repetitive seizures and were not treated with anticonvulsants. We believe that this study is the first to demonstrate the high seizure burden in PAIS using continuous multichannel EEG monitoring and is thus of significant and practical clinical importance.

The MCA is the most commonly involved artery for ischaemic infarction in term neonates (the posterior branch irrigates the occipital, temporal and posterior parietal areas, while the anterior branch irrigates the prefrontal, precentral, central and anterior parietal areas). [25] Clinical signs may not manifest if the motor cortical strip is not involved. [25] All neonates in our study had some degree of MCA involvement; at some timepoint a clinical correlate (often very subtle) was evident. Although typically neonates with PAIS are non-encephalopathic, [26] hypotonia, poor sucking reflex and irritability have been described. [27] Subtle seizures in our cohort involved mainly oral-buccal-lingual movements (four of six neonates); this is in line with other studies. [2], [28] In PAIS, autonomic dysfunction such as apnoeic spells [29], [30] has been reported in up to 36% of neonates; [31] only one neonate in our study presented with apnoea before any anticonvulsant administration. Other subtle seizures which have been previously described included eye blinking, vertical nystagmus and thumb adduction, [29] but multichannel EEG monitoring was not applied, thus the accuracy of these clinical signs is unknown. Our results support the suggestion for low threshold in initiating EEG monitoring when there is any suspicion of unusual movements which may be seizures.

To date, reported incidences of seizures in neonates with PAIS are mainly based on observation of neonatal behaviours, [22], [32] rather than on multichannel EEG which is the gold standard for accurate detection of neonatal seizures. [18], [33]–[35] Approximately 20% of neonatal seizures in term neonates are due to PAIS. [2] Conversely, while neonatal seizures have been noted in 26% of neonates with PAIS, [3] we believe these numbers could be much higher if detection of seizures is based on prolonged multichannel EEG monitoring. A limitation of our study is the small number of neonates with PAIS. In our cohort of neonates, all accept one neonate (case 2) was captured when they presented with hemiconvulsions before discharge shortly after birth in our 2 neonatal units. We only included neonates that presented with clear PAIS involving at most 2 arterial territories and who had continuous multichannel EEG monitoring as soon as possible after their presentation with seizures. While being monitored, these neonates with seizures showed asymmetrical characteristics on the EEG. In this period, other neonates would have presented but did not have continuous EEG monitoring undertaken. It is difficult to diagnose all neonates with PAIS in the neonatal period as the majority of term neonates affected by PAIS are asymptomatic; [1] appearing clinically well enough to be sent to the postnatal ward shortly after birth. In our 2 units, there is a policy of early maternal and neonatal discharge. Any neonate presenting with seizures after they were discharged would have been readmitted to regional paediatric hospitals, not the neonatal units. Even though our number of neonates with PAIS is small, we believe that the novelty here is having captured a number of neonates who had early and long duration of multichannel EEG monitoring.

In our study, EEG monitoring was initiated only after clinical seizures were observed in the first 3 days of life; we have shown that the age of first clinical seizure and first recorded EEG seizure [33 (17–42) and 36 (19–54) hours] were within 72 hours of age. This is current practice in most neonatal units as there are no existing early indicators to identify neonates with PAIS, hence it is possible that neonates with PAIS and electrographic-only seizures may have been missed during our recording period. Early EEG monitoring may have a role in providing an early indicator of PAIS, as early EEG from three hours after delivery has been shown to demonstrate occasional focal sharp waves over the infarcted region which became more frequent, complex and of higher amplitude in quiet sleep. [36]

In conclusion, EEG in neonates with PAIS demonstrated distinctive features in the background EEG and morphology of seizures. These features were present from very early after birth. Given the ease with which EEG monitoring can now be performed at the cotside, careful EEG analysis may prove very useful for early diagnosis of PAIS. For the first time, we have also quantified the seizure burden in neonates with PAIS using multichannel video-EEG. The majority of seizures in neonates with PAIS will escape detection without prolonged multichannel EEG monitoring.
